# EHMTI-0246. Lost productive time attributed to headache in a heavy-manufacturing workforce in Turkey

**DOI:** 10.1186/1129-2377-15-S1-B32

**Published:** 2014-09-18

**Authors:** M Selekler, G Gokmen, TJ Steiner

**Affiliations:** 1 Department of Neurology, Kocaeli University Faculty of Medicine, Kocaeli, Turkey; 2 Company Health Services, Ford Otomotiv Sanayi AS, Kocaeli, Turkey; 3 Department of Neuroscience, Norwegian University of Science and Technology, Trondheim, Norway

## Background

Headache disorders cause productivity losses through absenteeism and presenteeism. Productivity losses may be influenced as much by culture, social factors (employment levels) and the nature of the work as by frequency and severity of headache.

## Aim

To investigate productivity loss and its characteristics due to headache.

## Methods

We studied headache-attributed time losses in the workforce (n=7,200) of Ford Otomotiv Sanayi AS, a vehicle manufacturing company. Over one year, the HALT-30 questionnaire was administered to every employee during their routine annual health-check.

## Results

We obtained usable data from 5,916 employees (92.7% male, 7.3% female; mean age 32.5±5.4 years) among whom 1-month headache prevalence was 45.4%, with 896 (16.4% of the workforce) reporting headache-attributed productivity loss. Presenteeism greatly outweighed absenteeism (3,036 [94%] vs 190 mean total days/month). The nature of an employee’s work, from office and managerial through paint-house to heavy manufacturing (welder, assembler, press-metal worker), had insignificant impact on the probability of reporting productivity losses (range 15.2-18.8%) or on the mean loss per individual (range 2.8-3.6 days/month).

## Discussion

The lost productive time recorded was about 2.3% of all available time – a substantial penalty. It was surprising that the nature of work had so little influence, but it may be that the country’s economic state and unemployment rate, and the related social issues, were dominant factors. Supporting this was the finding that 94% of lost productivity was accounted for by presenteeism – largely hidden from the employer.

No conflict of interest.

